# Editorial: Digital Transformation of Animal Health Data: Proceedings of the AHEAD 2017 Workshop

**DOI:** 10.3389/fvets.2018.00111

**Published:** 2018-06-11

**Authors:** Flavie Vial, András Székács, Sinead Quealy

**Affiliations:** ^1^Epi-Connect, Skogås, Sweden; ^2^Agro-Environmental Research Institute, National Agricultural Research and Innovation Centre, Budapest, Hungary; ^3^VirtualVet, Waterford, Ireland

**Keywords:** digitisation, food animal production, antimicrobial usage data collection, agri-business models, real-time analysis, evidence-based policy

**Editorial on the Research Topic**

**Digital Transformation of Animal Health Data: Proceedings of the AHEAD 2017 Workshop**

Experts from government, farming, information and communications technology, policy and research convened on March 1st and 2nd 2017 in Exeter (UK) to create a roadmap to the digital transformation of animal health surveillance. The workshop, supported by the Organisation for Economic Co-operation and Development (OECD)’s Co-operative Research Programme on Biological Resource Management for Sustainable Agricultural Systems, was attended by representatives from 10 OECD countries (Australia, Belgium, Estonia, Hungary, Republic of Ireland, the Netherlands, Sweden, Switzerland, the United Kingdom and the United States of America)

Prof Toby Mottram’s opening words to the workshop, “We need a better picture of the creature we are trying to create”, resonated with all participants. In an attempt to sketch that picture, the structure of the workshop and of this editorial is very much aligned with the magic triangle for business models (1): Why? What? and How?

In this research topic, 21 authors contributed 9 articles (3 perspective pieces, 5 opinion papers and 1 review) arguing the paramount importance of collecting digital animal health data to strengthen our understanding of emerging issues including antimicrobial resistance; identifying the frameworks and tools required to support this digital revolution and proposing new societal and business models to profoundly change the way we all think about digital data.

## The “Why” of Animal Health Data Digitisation

In the EU, policy supports the reduction of antibiotic use, through the March 2016 new Animal Health Law (Regulation 2016/429), and a growing focus in the veterinary practice is to move away from blanket dosage of antibiotics, for example for mastitis. Significant and speedy improvements can take place, but only with coordinated actions supported by the entire value chain. Reducing the use of antibiotics is of massive societal importance, but changing on farm or veterinary methods requires thought and a user-centred approach. The most glaring and addressable challenge is the absence of near real-time data and information. AHEAD 2017 explored, in the context on EU Animal Health Law, how governments globally can benefit from increased digitisation in animal health.

Antimicrobial usage and resistance research data needs are explored in Magouras et al.; Pinto Ferreira. Many fairly simple questions still remain without clear answers: what quantity of antimicrobial drugs are used in veterinary medicine for different species? What are the social factors contributing to antimicrobial usage in the farming industry? How can we measure the association between antimicrobial usage and resistance? What environmental factors or contaminants accelerate antimicrobial resistance, what is the flow and fate of antimicrobial drugs and resistance into the environment and wildlife? In relation to the later, the review by Klátyik et al. brings to light the extent of the problem of residues of the active ingredients and adjuvants of veterinary drugs entering the food and the feed chains. The digital collection of data on veterinary drugs from production at pharmaceutical plants to their prescription by veterinarians and ultimately their usage on farm would provide elements of answers to all these questions. A strong call to action from industry stakeholders to see the wider value in early and digital data collection is made by Barrett.

## The “What” of Animal Health Data Digitisation

The sequence of processes required for evidence-based animal health decisions are delimited (Vial and Tedder) where the challenges to near real-time farm data analysis and interpretation are explored in more depth. These challenges include, but are not limited to, the weak adoption of standards and control vocabularies (for data interoperability), the need for data privacy and security, the need for modelling methods capable of handling high dimensionality and large sample size, and the need for context during output interpretation. Open global standards for use in the recording, storing, and sharing of data by the food and other industry sectors worldwide do exist and are presented (Bracken). If employed more widely along the animal production chain, these standards could make data capture at the point of treatment of the animal(s) and digital drug records a reality. Information technology systems accessible to decision makers working in the livestock industry have emerged, and Alawneh et al. presents one such system developed and used in New Zealand. Timely access and appropriate analysis of dairy herd productivity data is used to guide the allocation of resources to the most promising herd health interventions.

## The “How” of Animal Health Data Digitisation

Kärner explains how Estonian farmers, as all Estonian citizens, benefit from the usage of e-services. Estonian political and technical leadership laid the foundation for e-Estonia in the early 2000s on the principles of (1) decentralisation, (2) interconnectivity, (3) open platform and (4) open-ended process. Some of these principles are echoed in Lynch and Quealy’s participatory market model for the animal health industry. The animal health value chain has traditionally been a closed captive prescriptive market model built on transactional relationships and resulting in knowledge being siloed and in inefficient resource utilisation. Lynch and Quealy describe data ownership as “the most frequent roadblock” encountered when trying to engage with stakeholders in discussions around improved data capture and sharing. Today, this value chain is rapidly evolving with many new participants (e.g., feed and pharmaceutical companies) and the increasing capabilities of smart, connected products redefining the food-production industry. This context provides an opportunity to overcome these data tensions through the adoption of new business models to meet the needs of farmers, researchers, policy makers, and consumers.

This research topic draws attention to the fact that the digital transformation of animal health will require the involvement of stakeholders across several sectors and industries. All contributions make clear that a considerable number of frameworks, practices and tools already exist which can be extended to the animal health and agri-food industries. Researchers have demonstrated the advantages to implementing these as solutions to acknowledged challenges. While this digital transformation still appears to many as daunting, the advantages of such a transformation in data collection and data exchange would be enormous to all stakeholder groups (producers, consumers, pharmaceutical companies, food safety authorities, etc.) along the entire food chain and the wider scientific community

## Author Contributions

SQ and FV organised and ran the AHEAD 2017 workshop. FV and AS edited contributions to this research topic. All the authors were involved in writing this editorial and agree to its final version.

## Conflict of Interest Statement

The authors declare that the research was conducted in the absence of any commercial or financial relationships that could be construed as a potential conflict of interest.

## Disclaimer

The opinions expressed and arguments employed in this publication are the sole responsibility of the authors and do not necessarily reflect those of the OECD or of the governments of its Member countries.
